# Dr. Edw. Harrison

**Published:** 1838-07

**Authors:** 


### DR. EDWARD HARRISON.

On the 6th May, at Marlborough, while on his way to visit a patient. Dr. Harrison was born in 1766, and was consequently in his seventy-second year when he died. He was descended from an ancient family in Lancashire, where he was born. He studied at Edinburgh, where he took the degree of M.D. in 1784; his thesis being "De Opio." After graduation, he prosecuted his studies both in London and Paris, and finally settled at Horncastle, in Lincolnshire, where he practised thirty years. While here, he made himself conspicuous by strenuous efforts at medical reform, which originated in the best motives, but were not carried into effect with much discretion. He, however, left behind him more permanent memorials of his activity and benevolence in the Horncastle Dispensary, the Lincolnshire Benevolent Medical Society, and the Horncastle General Book-Club; of all which he was the founder. In 1817 he came to London, and from that period devoted himself almost exclusively to the treatment of spinal affections; on which he published, in 1827, a work entitled "Pathological and Practical Observations on Spinal Diseases." His views were peculiar, and not generally sanctioned by pathologists; and his practice was obnoxious to the charge of attending too much to the local deformity, and too little to the constitutional debility in which this originated: the consequence was, that many of his patients who became straight, under his manipulations and prescribed recumbency, soon relapsed into their original crookedness, on taking their accustomed exercise. Dr. Harrison died rich, and bequeathed considerable sums to various charities.

